# Role and Mechanism of Keap1/Nrf2 Signaling Pathway in the Regulation of Autophagy in Alleviating Pulmonary Fibrosis

**DOI:** 10.1155/2022/3564871

**Published:** 2022-07-18

**Authors:** Zhaoxing Dong, E. Gao Yin, Meijuan Yang, Xiaoyuan Zhao, Jing Li, Wen Lei

**Affiliations:** ^1^Department of Respiratory Medicine, Hua Mei Hospital, University of Chinese Academy of Sciences, Ningbo Institute of Life and Health Industry, University of Chinese Academy of Sciences, Ningbo, 315010, China; ^2^The Fist Ward, Department of Pulmonary and Critical Care Medicine, The Second Affiliated Hospital of Kunming Medical University, Kunming, 650101, China

## Abstract

A variety of internal and external lung diseases may eventually lead to pulmonary fibrosis, and insufficient autophagy is closely related to pulmonary fibrosis. This research is aimed to explore the mechanism of autophagy to alleviate pulmonary fibrosis. Then, a mouse model of pulmonary fibrosis induced by boromycin and histopathological lesions of the lungs of mice were observed by HE staining, which Masson staining assessed the degree of fibrosis in the lung tissue by detecting the expression of hydroxyproline in the tissue. RT-qPCR and western blotting were used to detect the levels of autophagy and Keap1/Nrf2 signaling pathway-related proteins. It was proved that autophagy-related proteins MAP1LC3(LC3) and Beclin 1 were decreased in mice with pulmonary fibrosis, while the expression of p62 was increased. Mice with pulmonary fibrosis worsened after injection of a 3-MA autophagy inhibitor, while injection of autophagy activation of rapamycin agent promoted Nrf2 nuclear mobilization. In a word, autophagy relieves pulmonary fibrosis through the activation of the Keap1/Nrf2 signaling pathway.

## 1. Introduction

Pulmonary fibrosis is an inflammatory disease characterized by the proliferation of fibroblasts and the accumulation of extracellular matrix accompanied by inflammatory damage [1]. Most patients with pulmonary fibrosis have an unknown etiology (idiopathic). Idiopathic pulmonary fibrosis (IPF) with pulmonary fibrosis as the main manifestation is the most common disease, which can lead to progressive loss of lung function [2]. Pulmonary fibrosis seriously affects human respiratory function. After normal lung tissue is replaced by fibrotic tissue, respiratory function will be significantly reduced [1]. The morbidity and mortality of pulmonary fibrosis are increasing year by year and the average survival time after diagnosis is only 2.8 years. These claims have shown a poor prognosis clinically [3, 4]. Therefore, it is of great significance to explore the mechanism of pulmonary fibrosis.

Autophagy is a process of engulfing one's own cytoplasmic proteins or organelles and coating them into vesicles and fusing with lysosomes to form autophagic lysosomes and degrading the contents it encapsulates. It is a conservative decomposition of cells' metabolic needs that can also achieve the renewal of organelles [5]. In the literature, various mechanisms have found that autophagy plays an important role in the regulation of lung diseases, especially in diseases of pulmonary fibrosis [6]. The proliferation of fibroblasts is a major feature of pulmonary fibrosis and autophagy can maintain the normal life and progression of lung fibroblasts. Additionally, transforming growth factor-1 (TGF-*β*1) can induce fibroblasts to myofibroblasts differentiation, which leads to pulmonary fibrosis, mainly through the inhibition of autophagy, indicating that the intervention of autophagy can be an effective measure to prevent pulmonary fibrosis [7]. The direct impact of autophagy on pulmonary fibrosis has also been proved, for example, bleomycin-induced pulmonary fibrosis can be improved by inhibiting apoptosis of pulmonary epithelial cells through autophagy. Likewise, the destruction of inhibition of cellular protease after autophagy blocking can induce the occurrence of interstitial lung disease [8, 9]. Therefore, exploring the mechanism of autophagy regulating the progression of pulmonary fiber can provide a new treatment idea for alleviating pulmonary fibrosis.

Keap1-Nrf2 is one of the important mechanisms of cell defense against oxidative stress damage. After the Keap1-Nrf2 signaling pathway is activated, the nuclear transcription factor Nrf2 enters the nucleus and activates the transcription of a variety of antioxidant genes, thereby reducing cell damage caused by ROS [10–12]. Studies have found that the Keap1-Nrf2 signaling pathway has an important effect on autophagy, and the expression of autophagy gene will also decrease after Nrf2 is knocked out [13]. In addition to it, induction of autophagy can be regulated by the systemic level feedback of Nrf2 based on oxidative stress response [14]. Meanwhile, studies have shown that Keap1-Nrf2 is associated with pulmonary fibrosis, and the protective effect of p65 in pulmonary fibrosis can be reversed by Nrf2 knockout [15]. The activation of the Keap1-Nrf2 feedback loop can promote the antioxidant response of autophagy to improve the harmful effects of excessive oxidative stress. In this paper, we have explored the mechanism of autophagy in pulmonary fibrosis and to clarify the mechanism of the interaction between Keap1/Nrf2 signaling pathway and autophagy in pulmonary fibrosis. For this purpose, various experimental studies, specifically on mouse dataset, were performed to verify the expected outcome and claims of the proposed model.

## 2. Materials and Methods

In this section, a detailed description of the experimental studies, their possible setup, and approval from the concerned authority (if needed and applicable) is provided. Additionally, status of the animal, mouse in this case, and the effects of the proposed solution of their status are described in detail.

### 2.1. Animals

Animal experiments were approved by the Animal Experiment Ethics Review Committee of Kunming Medical University (Lot Number: kmmu2021756). Forty-eight male, which were 8-week-old C57BL/6 mice, weighing about 18–22 g and SPF grade. These mice were fed for seven days to adapt to the environment and then randomly divided into 4 groups such that every group has 12 mice.

Grouping: (i) Sham operation group (Sham), (ii) bleomycin (BLM) group, (iii) BLM + autophagy inhibitor (3-MA) group, and (iv) BLM + autophagy activator rapamycin (RAPA) group. Initially, twelve (12) hours before the experiments, mice were forced to fast (as no food was provided) and gave them only water. All experimental mice were anesthetized with 2% sodium pentobarbital and administered by intraperitoneal injection of 0.056 mg/kg. After the mice did not respond, they were placed on a fixed plate and the limbs and head of the mice were fixed. Skin and sterilize sharp scissors are used to cut a small vertical 0.5 cm wound up in the center of the neck. The cortex and muscle layer were peeled off to expose the trachea and used a 1 ml syringe to push the BLM (2.5 mg/kg) along the lower edge of the cartilage ring and injected into the airway (0.9% sodium chloride injection in the Sham group), and then turned the test bench. The BLM (0.9% sodium chloride injection in the Sham group) was let to reach the small airways in the lungs, arrange, and suture the wound. To prevent wound infection, mice were given amikacin lotion on the wound 3 days after the operation. On the basis of the successful construction of pulmonary fibrosis mice, subsequent BLM mice were injected with autophagy inhibitor (3-MA) and autophagy activator (rapamycin) for intervention to complete various tests in subsequent experiments.

### 2.2. Culture of Pulmonary Fibrosis Cells (PFC)

Lung tissue was extracted from a 10-week-old male mice in the aseptic state, cut into 0.5–1 mm^3^ tissue blocks, sterilized with PBS, and rinsed twice to remove blood and floating connective tissue. The tissue mass was transferred into a 50 mL centrifuge tube and 0.25% trypsin 5 mL was added. The tissue mass was digested at 37°C for 5–10 min and shake the tube gently by hand during digestion. Gently blown and discarded the upper suspension, added trypsin 5 mL to the tissue mass for further digestion for 5–10 min, centrifuged, collected the supernatant and added it to Dulbecco's Modified Eagle's Medium (DMEM) containing 20% BSA to stop digestion, and blown and collected the supernatant. These steps were repeated three times. The collected supernatant was centrifuged at 1000 r/min for 10 min. The supernatant was discarded and added with 10% fetal bovine serum(FBS) DMEM medium was blown away and inoculated in the culture flask, placed in the incubator (5% CO_2_, 37°C) for 2 h, discarded the supernatant, replaced with fresh medium, and continued the culture.

### 2.3. RT-qPCR

After collecting mouse lung tissue or lung fibrocytes, the total RNA was extracted using TRIzol reagent (Invitrogen; Thermo Fisher Scientific Inc.) according to the manufacturer's instruction. Then, cDNA was synthesized using reverse transcription kit (Bio-Rad Laboratories Inc.). Finally, cDNA was used as a template to detect RT-qPCR reaction with PCR kit (Takara Bio Inc.). The results were obtained, and the method of 2^−△△Ct^ was used to analyze the relative expression level.

### 2.4. Western Blotting

Initially, (i) extracted the protein of cells and tissues, (ii) used the BCA kit to determine the protein concentration, (iii) prepared a polyacrylamide gel of the corresponding concentration according to the molecular weight of the protein, and (iv) adjusted the sample amount according to the protein concentration. After the electrophoresis was completed, the protein was transferred to the PVDF membrane, which was pretreated with formaldehyde (transferred conditions were 4°C, 90 mA, and 1.5 h). Blocked with 5% skimmed milk powder for 1 h, and then added the (i) diluted primary antibody, (ii) translight chain 3 (LC3) antibody (1 : 1000 dilution), (iii) Beclin1 antibody (1 : 500 dilution), (iv) p62 antibody (1 : 2000 dilution), (v) Keap1antibody (1 : 2000 dilution), and (vi) Nrf2 antibody (1 : 1000 dilution) at 4°C overnight. Added HRP-labeled secondary antibody and incubated at room temperature for 1 h. ECL color development, gel imaging system imaging analysis, semiquantitative determination of expression level, and ImageJ analysis of band gray value were performed.

### 2.5. Hematoxylin and Eosin (H&E) Staining

The slices were routinely dewaxed, which was (i) washed with water for 10 s, (ii) washed with hematoxylin dye solution for 10 min, (iii) rinsed with running water for 1 min, (iv) washed with 1% hydrochloric acid alcohol for 10 s, (v) rinsed in running water for 1 min, (vi) washed with warm water returned to blue for 1 min, (vii) put in eosin dye solution for 30 s, and (viii) washed in water for 10 s. Gradient alcohol dehydration, transparent xylene, mounting with neutral gum, and observed the cell morphology under a fluorescence microscope and took pictures and recorded.

### 2.6. Masson Staining

After the lung tissue was embedded and fixed in paraffin, the tissue section was stained according to the instructions of the Masson staining kit, and then the staining of the section was observed under a microscope.

### 2.7. Determination of Hydroxyproline (HYP)

After grinding the lung tissue into a homogenate shape, detected the content of HYP in the homogenate according to the instructions of the hydroxyproline detection kit.

### 2.8. Statistical Analysis

GraphPad Prism 8 software was used to draw relevant statistics and SPSS19.0 was used for statistical analysis. Data differences between groups were analyzed by *t*-test (Student's test) and one-way ANOVA where *P* < 0.05 indicated a significant difference and *P* < 0.01 indicated a very significant difference, respectively.

## 3. Experimental Results and Observations

In this section, various observations and the experimental study are reported and described in detail how effective the proposed approach is. Each experiment was performed using sophisticated procedure such as safety of both animals and scientists. The experimental results and observations of various groups are compared against each other, which were carried out under similar environmental conditions.

### 3.1. Expression of Autophagy-Associated Proteins and Keap1/Nrf2 Pathway-Associated Proteins in BLM-Induced PF

**Figure 1:**
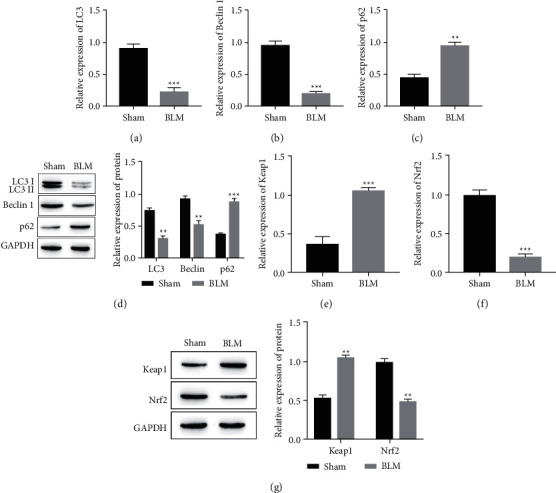
The expression levels of autophagy and KEAP1/Nrf2 signaling pathway-related proteins in pulmonary fibrosis mouse models. (a) The expression level of LC3 was detected by RT-qPCR. ^*∗∗∗*^*P* < 0.001 vs. Sham group. (b) The expression level of Beclin 1 was detected by RT-qPCR. ^*∗∗∗*^*P* < 0.001 vs. Sham group. (c) The expression level of p62 was detected by RT-qPCR. ^*∗∗*^*P* < 0.01 vs. Sham group. (d) The expression levels of autophagy-related proteins of LC3, Beclin 1, and p62 by Western blotting assay. ^*∗∗*^*P* < 0.01, ^*∗∗∗*^*P* < 0.001 vs. Sham group. (e) The expression level of KEAP1 was detected by RT-qPCR. ^*∗∗∗*^*P* < 0.001 vs. Sham group. (f) The expression level of Nrf2 was detected by RT-qPCR. ^*∗∗∗*^*P* < 0.001 vs. Sham group. The expression levels of KEAP1/Nrf2 signaling pathway-related proteins of KEAP1 and Nrf2 by Western blotting assay. ^*∗∗*^*P* < 0.01 vs. Sham group.

BLM-induced lung fibrosis mouse lung tissue was taken as a sample to detect autophagy and Keap1/Nrf2 pathway-related protein expression. Comparison with mice in the Sham group, RT-qPCR, and Western Blot tests, showed a significant decrease in the expression of autophagy-related proteins LC3 and Beclin 1 in BLM mice (Figures 1(a), 1(b), and 1(d), *P* < 0.05) and a significant increase of p62 (Figures 1(c) and 1(d), *P* < 0.05). At the same time, RT-qPCR and Western Blot detection of proteins related to the Keap1/Nrf2 signaling pathway showed that the expression of Keap1 was significantly increased in BLM mice (Figures 1(e) and 1(g), *P* < 0.05), while the expression of Nrf2 was significantly decreased than that of the Sham group (Figures 1(f), 1(g), *P* < 0.05).

### 3.2. Autophagy Attenuates the Pathological Damage and Collagen Accumulation of Pulmonary Fibrosis

**Figure 2:**
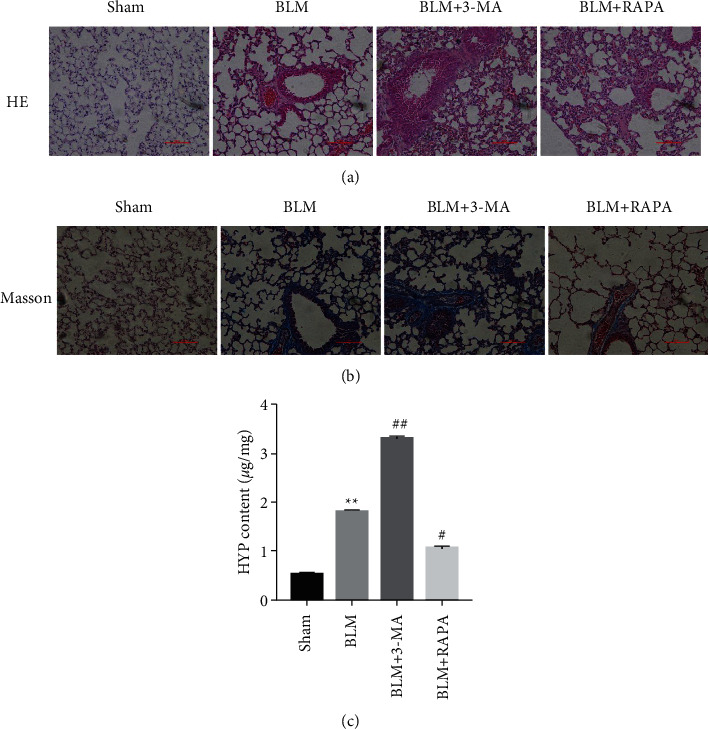
The effect of autophagy on BLM-induced PF. (a) HE staining to detect histopathological changes. (b) Masson staining to detect collagen accumulation. (c) The kit detects the content of HYP in lung tissue. ^*∗∗*^*P* < 0.01 vs. Sham group. ^#^*P* < 0.05, ^##^*P* < 0.01 vs. BLM group.

HE staining and Masson staining were performed on mouse lung tissue to observe pathological changes. The results of HE staining showed that the lung tissue structure of the BLM-perfused mice was severely damaged and the alveoli were significantly reduced and deformed (Figure 2(a)). The results of Masson staining showed that the lung tissue of the mice perfused with BLM was disordered and there was a large amount of collagen deposition (Figure 2(b)). However, treatment with autophagy activator RAPA partially restored BLM-induced alveolar structural damage and inhibited BLM-induced collagen deposition (Figures 2(a) and 2(b)). The autophagy inhibitor 3-MA has the opposite effect (Figures 2(a) and 2(b)). In addition, by detecting the content of HYP in the lung tissue, it was found that compared with the Sham group (0.52 ± 0.02 *μ*g/mg), the content of HYP in the lung tissue of the BLM perfusion (1.81 ± 0.03 *μ*g/mg) group was significantly increased (Figure 2(c)). Similarly, autophagy inhibitor 3-MA further increased the content of HYP while autophagy activator RAPA reduced it (Figure 2(c)).

### 3.3. The Expression of Autophagy-Related Proteins and Keap1/Nrf2 Pathway-Related Proteins in Lung Fibroblasts Cell

**Figure 3:**
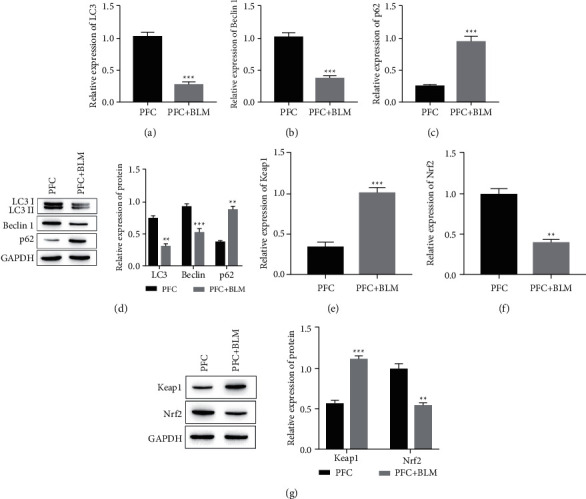
The expression levels of autophagy and KEAP1/Nrf2 signaling pathway-related proteins in PFC cells. (a) The expression level of LC3 was detected by RT-qPCR. ^*∗∗∗*^*P* < 0.001 vs. PFC + BLM group. (b) The expression level of Beclin 1 was detected by RT-qPCR. ^*∗∗∗*^*P* < 0.001 vs. PFC + BLM group. (c) The expression level of p62 was detected by RT-qPCR. ^*∗∗*^*P* < 0.01 vs. PFC + BLM group. (d) The expression levels of autophagy related proteins of LC3, Beclin 1, and p62 by Western blotting assay. ^*∗∗*^*P* < 0.01, ^*∗∗∗*^*P* < 0.001 vs. PFC + BLM group. (e) The expression level of KEAP1 was detected by RT-qPCR. ^*∗∗∗*^*P* < 0.001 vs. PFC + BLM group. (f) The expression level of Nrf2 was detected by RT-qPCR. ^*∗∗*^*P* < 0.01 vs. PFC + BLM. The expression levels of KEAP1/Nrf2 signaling pathway-related proteins of KEAP1 and Nrf2 by Western blotting assay. ^*∗∗*^*P* < 0.01*vs*. PFC + BLM group.

Comparison with PFC group, RT-qPCR, and Western Blot tests showed a significant decrease in the expression of autophagy-related proteins LC3 and Beclin 1 in the PFC+BLM group (Figures 3(a), 3(b), and 3(d), *P* < 0.05) and a significant increase of p62 (Figures 3(c), and 3(d), *P* < 0.05). This result is consistent with the detection results in the BLM-induced mouse model of PF. The expression of proteins related to the Keap1/Nrf2 signaling pathway was also detected by RT-qPCR. The results showed that the expression of Keap1 was significantly increased in PFC + BLM group (Figures 3(e), 3(g), *P* < 0.05), while the expression of Nrf2 was significantly decreased, compared with the PFC group (Figure 3(f), 3(g), *P* < 0.05).

### 3.4. Effects of Autophagy on Proliferation and Apoptosis of PFC Cells

**Figure 4:**
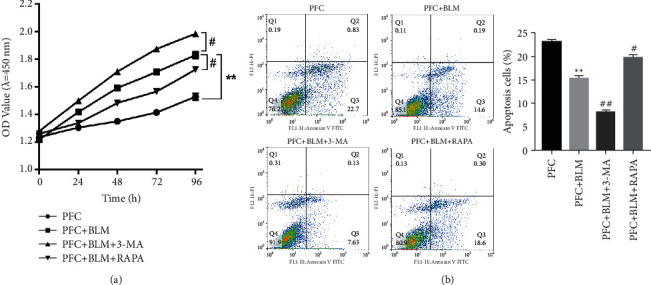
Effects of autophagy on proliferation and apoptosis of PFC cells. (a) The cell proliferation of PFC cells were examined by CCK-8 assay. ^*∗∗*^*P* < 0.01 vs. PFC group. ^#^*P* < 0.05 vs. PFC + BLM group. (b) The cell apoptosis of PFC cells were examined by CCK-8 assay. ^*∗∗*^*P* < 0.01 vs. PFC group. ^#^*P* < 0.05, ^##^*P* < 0.01*vs.* PFC + BLM group.

CCK-8 test results showed that compared with the PFC group, the proliferation activity of cells in the PFC + BLM group was significantly increased (Figure 4(a)). However, when the autophagy activator was applied, the proliferation activity of PFC cells was attenuated, while the autophagy inhibitor had the opposite effect, which could further promote the proliferation activity of cells (Figure 4(a)). The results of flow cytometry detection of apoptosis showed that compared with the PFC group, the apoptosis in the PFC + BLM group was significantly declined, and the autophagy activator could enhance the occurrence of apoptosis in the PFC, while the autophagy inhibitor could further reduce the occurrence of apoptosis in the PFC cells (Figure 4(b)).

### 3.5. Autophagy Affects Cell Proliferation and Apoptosis through Keap1/Nrf2 Signaling Pathway

**Figure 5:**
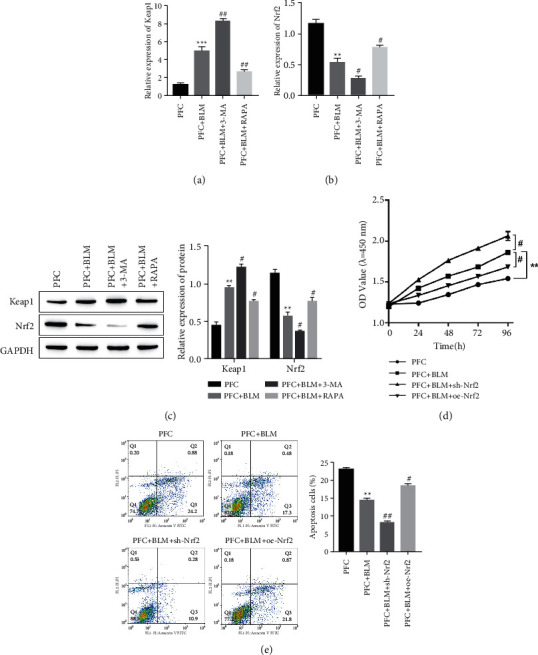
Autophagy affects cell proliferation and apoptosis through KEAP1/Nrf2 signaling pathway. (a) The expression levels of KEAP1 by RT-qPCR. ^*∗∗∗*^*P* < 0.001 vs. PFC group. ^##^*P* < 0.01 vs. PFC + BLM group. (b) The expression levels of Nrf2 by RT-qPCR. ^*∗∗*^*P* < 0.01 vs. PFC group. ^#^*P* < 0.05 vs. PFC + BLM group. (c) The expression levels of KEAP1 and Nrf2 by Western blotting assay. ^*∗∗*^*P* < 0.01 vs. PFC group. ^#^*P* < 0.05 vs. PFC + BLM group. (d) The cell proliferation of PFC cells were examined by CCK-8 assay. ^*∗∗*^*P* < 0.01 vs. PFC group. ^#^*P* < 0.05 vs. PFC + BLM group. (e) The cell apoptosis of PFC cells were examined by CCK-8 assay. ^*∗∗*^*P* < 0.01 vs. PFC group. ^#^*P* < 0.05, ^##^*P* < 0.01 vs. PFC + BLM group.

RT-qPCR and Western Blot results showed that compared with the PFC group, the expression of Keap1 was significantly higher in the PFC + BLM group, and the expression of Nrf2 was significantly decreased in the PFC + BLM group, but the application of autophagy activator of RAPA could significantly reduce the expression of Keap1 and increase the expression of Nrf2 in the PFC + BLM group, while the autophagy inhibitor of 3-MA could further promote the expression of Keap1 and reduce the expression of Nrf2 (Figures 5(a)–5(c)). CCK-8 test results showed that compared with the PFC group, the proliferation activity of cells in the PFC + BLM group was significantly increased, but the proliferation activity of cells was significantly decreased after the overexpression of Nrf2, and the opposite result was obtained after the knockdown of Nrf2 (Figure 5(d)). The results of flow cytometry detection of cell apoptosis showed that compared with the PFC group, the apoptosis of the PFC + BLM group was significantly decreased. When the autophagy activator was added, the occurrence of cell apoptosis could be promoted, while when the autophagy inhibitor was added, the occurrence of cell apoptosis could be further weakened (Figure 5(e)).

## 4. Performance Evaluation: General Discussion

Pulmonary fibrosis leads to progressive fibrosis and loss of lung function, making it a progressive and fatal lung disease. The occurrence of pulmonary fibrosis will have obvious tissue pathological features such as fibroblast proliferation, cell inflammation of the deposited interstitium of the outer matrix, or collapse of alveolar cells [16]. At present, research on the treatment of pulmonary fibrosis is vigorously carried out, but the road to successful treatment is still very long, and the proposal of new treatment strategies is also very necessary [17]. Autophagy is proposed as an effective intervention for the prevention and improvement of pulmonary fibrosis. The proposed research work shows that autophagy can alleviate the occurrence of pulmonary fibrosis by activating the Keap1/Nrf2 signaling pathway, and autophagy activators can probably effectively slow down lung injury from BLM-induced pulmonary fibrosis mice, as well as increase the apoptosis of lung fibrotic cells and inhibit the proliferation of lung fibrotic cells, while autophagy inhibitors produce the opposite result.

BLM is used as an antibiotic in the treatment of cancer, but it can produce dangerous side effects, namely, bleomycin pulmonary toxicity, which can lead to loss of lung function [18]. Studies have shown that 2–46% of patients treated with this drug will be accompanied by the side effect of pulmonary toxicity [19–21], and the mortality rate of BLT is 1-2% [22, 23]. Patients who receive bleomycin during surgery and require oxygen support are also prone to pulmonary toxicity in a state of hypoxia, which may be caused by the induction of superoxide anion [24]. At the same time, in the state of hyperoxia, oxygen-free radicals can inactivate antioxidant enzymes and cause the death of oxygen-sensitive cells, leading to the death of alveolar cells [25]. The pulmonary toxicity of bleomycin can cause the destruction of lung structure and cause pulmonary fibrosis, specifically by affecting the transcription of fibroblasts and the increase of collagen production or the increase of hydroxyproline level [26] In this study, in mice with bleomycin-induced pulmonary fibrosis, we found that the alveolar structure of the mice was damaged, the alveolar septum was widened, a large amount of collagen deposition appeared, and the content of hydroxyproline increased.

Autophagy is a self-protection mechanism of cells in a bad environment or external stimuli such as starvation, hypoxia, and DNA damage [27]. When autophagy occurs, some proteins play a key role, and Beclin1 and LC3 are two typical autophagy markers [28]. Among them, p62 also plays an important role in autophagy, p62 is an important indicator of the dynamic process of autophagy, and p62 can bind to the autophagy substrate LC3. By detecting the expression of LC3II and p62 in the lung tissue of IPF patients, it was found that the down-regulation of LC3II expression and the up-regulation of p62 expression proved that there is insufficient autophagy in the lung tissue of IPF patients [29]. In addition, it was also found that compared with normal lung, the expression of Beclin1 in IPF lung fibroblasts was down-regulated and LC3II was inhibited [30, 31]. Similarly, in this study, we found that in BLM-induced pulmonary fibrosis mice, the expressions of LC3 and Beclin1 were significantly decreased, while the expression of p62 was significantly increased, which was consistent with the results of previous studies and also showed the important role of autophagy in the progression of pulmonary fibrosis.

As an inducible transcription factor, Nrf2 can play an adaptive protective role in the state of cellular oxidation and protein toxic stress, thereby maintaining the continuation of redox signals [32]. In addition, the regulatory role of Nrf2 in the inflammatory response has also been reported [33]. Similarly, Nrf2 also plays an important role in pulmonary fibrosis, and it can participate in the fine-tuning of autophagy in response to oxidative stress levels [14]. The role of Nrf2 as a “master regulator” in the antioxidant response has been proven to play a key role in bleomycin-induced pulmonary fibrosis, and it has also become a biomarker and potential therapeutic target [34]. The role of Keap1/Nrf2 signaling pathway has received extensive attention. Studies have found that the activation of Keap1/Nrf2 signaling pathway can regulate autophagy and apoptosis regulated by ROS [35, 36], and the feedback loop of Keap1/Nrf2 can be maintained by p62 [37]. In this study, we have found that the expression of Keap1 and Nrf2 are abnormal in mouse models of pulmonary fibrosis and pulmonary fibrosis cells, that is, Keap1 is highly expressed and Nrf2 is low, indicating that the Keap1/Nrf2 signaling pathway is affected by lung fibrosis. In this study, it was observed that autophagy activators can reverse the abnormal expression of Keap1 and Nrf2 and activate the Keap1/Nrf2 pathway. Autophagy inhibitors have achieved the opposite result in the experimental setup.

## 5. Conclusions

In this paper, we have explored the mechanism of autophagy to alleviate pulmonary fibrosis. Formation of pulmonary fibrosis mouse model was induced by Boromycin and observed lung histopathological lesions in mice by HE staining, which shows the degree of fibrosis in tissue by Masson staining and detecting hydroxyproline expression in lung tissue. RT-qPCR and western blotting detected autophagy and Keap1/Nrf2 signaling pathway-related proteins. Furthermore, we have found that expression of LC3, Beclin 1, and Nrf2 are decreased in PF mouse model and cells. The expression of p62 and Keap1 are increased. Autophagy can reduce BLM-induced alveolar structural damage and inhibit the occurrence of collagen deposition. Autophagy alleviating pulmonary fibrosis through activation of Keap1/Nrf2 signaling pathway. This study provides a new treatment strategy for PF.

In future, we are eager to explore and investigate the expected performance of the proposed solution and perform experiments on human beings, preferably volunteers, instead of mice.

## Data Availability

The authors confirm that the data supporting the findings of this study are available within the article.
